# Genome-wide identification and functional prediction of long non-coding RNAs in plastic growth in *Alternanthera philoxeroides*

**DOI:** 10.1016/j.isci.2025.113488

**Published:** 2025-09-02

**Authors:** Ruiyi Qiu, Qianqian Hu, Wenbo Liu, Meiquan Qiu, Chuanwei Yang, Binglian Zheng, Ji Yang

**Affiliations:** 1 State Key Laboratory of Wetland Conservation and Restoration, National Observations and Research Station for Wetland Ecosystems of the Yangtze Estuary, Ministry of Education Key Laboratory for Biodiversity Science and Ecological Engineering, and Institute of Eco-Chongming, Fudan University, Shanghai, China; 2 State Key Laboratory of Genetic Engineering, Ministry of Education Key Laboratory of Biodiversity Sciences and Ecological Engineering, Institute of Plant Biology, School of Life Sciences, Fudan University, Shanghai 200438, China; 3 Shanghai Key Laboratory of Plant Functional Genomics and Resources, Shanghai Chenshan Botanical Garden, Shanghai, China

**Keywords:** Natural sciences, Plant bioinformatics, Plant biology, Plant physiology, Plant evolution

## Abstract

Plants have a remarkable ability to alter their growth and development to adapt to fluctuating environments. Epigenetics plays a pivotal role in regulating plastic phenotypes. Based on long-read PacBio sequencing and time-course gene expression profiling, a genome-wide survey and functional prediction of lncRNAs potentially involved in submergence-induced plastic growth was conducted in *A. philoxeroides*. A total of 141 submergence-responsive lncRNAs were identified, with their *cis*- and *trans*-targeting genes being predicted. Functional annotation and validation of lncRNA targets indicated the significant roles of *lncRNA14247*, *lncRNA13847*, and *lncRNA12385* on phytohormone homeostasis by mediating crosstalk between various phytohormones upon submergence, and thereby impacting plastic development. The results also unveiled the potential roles of *lncRNA13847* and *lncRNA7566* in mediating submergence-induced growth alterations by targeting light signal components, such as PIFs. These findings highlighted the significance of lncRNAs in submergence responses and provided insights into the molecular underpinnings of phenotypic plasticity in *A. philoxeroides*.

## Highlights

• A total of 141 submergence-responsive *cis-* and *trans-*acting lncRNAs were identified
• *LncRNA14247*, *lncRNA13847*, and *lncRNA12385* are involved in phytohormone homeostasis
• *LncRNA13847* and *lncRNA7566* promote plastic growth by regulating light signaling

## Graphical abstract

**Figure:**
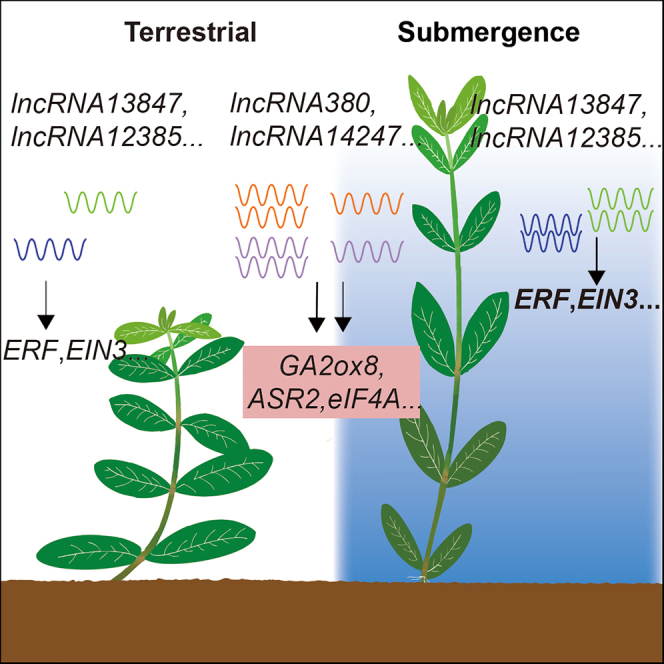

## Introduction

Long non-coding RNAs (lncRNAs) are a class of RNA transcripts exceeding 200 nucleotides in length and lacking effective open reading frame (ORF) or coding potential.^1^^,^^2^ Based on their genomic location and the product orientation of the DNA strand, lncRNAs are categorized into intergenic, intronic, sense, anti-sense, and bidirectional.^3^ Since the first discovery of lncRNAs in *Glycine max*, regulatory lncRNAs have been characterized in many plants.^4^ There is increasing evidence that lncRNAs, as key epigenetic regulators, play crucial roles in plant growth, reproduction, metabolism, and stress responses to biotic and abiotic factors.^5^^,^^6^^,^^7^^,^^8^ For instance, the lncRNA COLDAIR mediates the vernalization-induced repression of *FLC* in *Arabidopsis* through chromatin remodeling,^9^ while APOLO controls multiple aspects of root development by integrating endogenous and exogenous signals.^10^ Survey of high throughput RNA-Seq data has revealed that lncRNAs are involved not only in morphogenesis, but also in stress response in wheat (*Triticum aestivum*). In grapes, lncRNAs have been shown to act as regulatory players of responses to powdery and downy mildew infection.^11^ Recent advances in high-throughput RNA sequencing (RNA-seq) have facilitated the genome-wide identification of stress-responsive lncRNAs in different species. For example, 3,738 lncRNAs have been identified in *Cucumis sativus*, and 303 of which were differentially expressed in response to waterlogging.^12^ A study combining third-generation sequencing with high-throughput RNA-seq identified 829 lncRNAs in *Populus simonii*. Of them, 21 responded to heat stress.^13^ Of the 7,743 lncRNAs functionally annotated in wheat, about 29% lncRNAs were found to be responsive to heat, drought, and their combination stress, and around 37% lncRNAs were involved in response to salt stress.^14^ Target genes can be regulated by lncRNAs via either *cis*- or *trans-*acting processes, where they may be located nearby or distantly in the genome.^15^ Diverse mechanisms of lncRNA function have been reported, including chromatin modification, transcriptional interference, and post-transcriptional regulation.^3^^,^^15^

Plants, as sessile organisms, have a remarkable ability to alter their growth and development to adapt to fluctuating environments rapidly without undergoing genetic variation.^16^^,^^17^ The development of phenotypically plastic traits in response to changing environments is largely based on changes in gene expression, which is often mediated by epigenetic mechanisms.^18^^,^^19^^,^^20^^,^^21^^,^^22^^,^^23^ The roles for DNA methylation in phenotypic plasticity have been extensively described, showing that environmentally induced changes in DNA methylation and phenotype are strong in plants.^24^^,^^25^ DNA methylation may also facilitate the establishment and spread of invasive species by increasing phenotypic variation and plasticity.^26^ The pivotal role of microRNAs in governing plastic behavior during development, such as phase change and plant architecture, has been widely studied as well,^27^^,^^28^^,^^29^ including their contribution to successful biological invasions.^26^ Compared with DNA methylation and microRNAs, evidence in support of lncRNA-driven phenotypic plasticity and the success of invasive species are sparse, though lncRNAs have received significant attention as modulators of stress responses in plants. Uncovering the extent to which phenotypic plasticity is explained by lncRNA functions has implications not only for developing climate-resilient crops to ensure food security in the context of global climate change and frequent extreme weather events,^30^ but also for dissecting mechanisms of invasion because high phenotypic plasticity can facilitate the rapid colonization of new habitats, augmenting the invasive potential of alien species.^31^

*Alternanthera philoxeroides*, a perennial weed originating from South America, has currently spread worldwide including China.^32^^,^^33^ Previous studies have shown that the invasive populations of *A. philoxeroides* exhibit low genetic diversity in China, as the plants barely produce viable seeds and instead rely on vegetative regeneration to propagate.^34^ Nevertheless, the plants of *A. philoxeroides* can thrive in contrasting hydrological habitats, ranging from aquatic environments such as rivers and ponds to dry lands, and display environment-dependent phenotypic variations.^35^ The plants growing in aquatic habitats often exhibit significant increases in internode length and diameter, enhanced aerenchyma formation, and other morphological and physiological variations.^32^^,^^33^^,^^35^ Low genetic diversity and high phenotypic plasticity render *A. philoxeroides* an excellent model for elucidating the epigenetic mechanisms of phenotypic plasticity. Our previous studies have revealed rapid genome-wide alterations in DNA methylation^36^ and gene expression reaction norms^37^ of *A. philoxeroides* under varying hydrological conditions. Dynamic microRNA expression patterns have also been identified in contrasting hydrological habitats. MicroRNA-mRNA pairs associated with plastic internode elongation were discovered to participate in gibberellic acid (GA) synthesis and abscisic acid (ABA) signaling.^38^ These studies provided evidence linking epigenetic regulation to the plastic response of *A. philoxeroides* to changing environmental conditions.

In this study, we aimed to identify the lncRNAs participating in the regulation of submergence-induced plastic variations in *A. philoxeroides* by integrating third-generation long-read PacBio SMRT sequencing and time-series Illumina RNA-seq data. The *cis*- and *trans*-target genes of tissue-specific submergence-responsive lncRNAs were predicted based on the location relationship between lncRNAs and mRNAs and co-expression patterns. These results would provide insights into the potential role of lncRNAs in regulating plastic developmental responses to submergence and enrich our understanding of the epigenetic regulation underpinning phenotypic plasticity and invasiveness.

## Results

### Morphological and transcriptomic responses to submergence in A. philoxeroides

**Figure 1:**
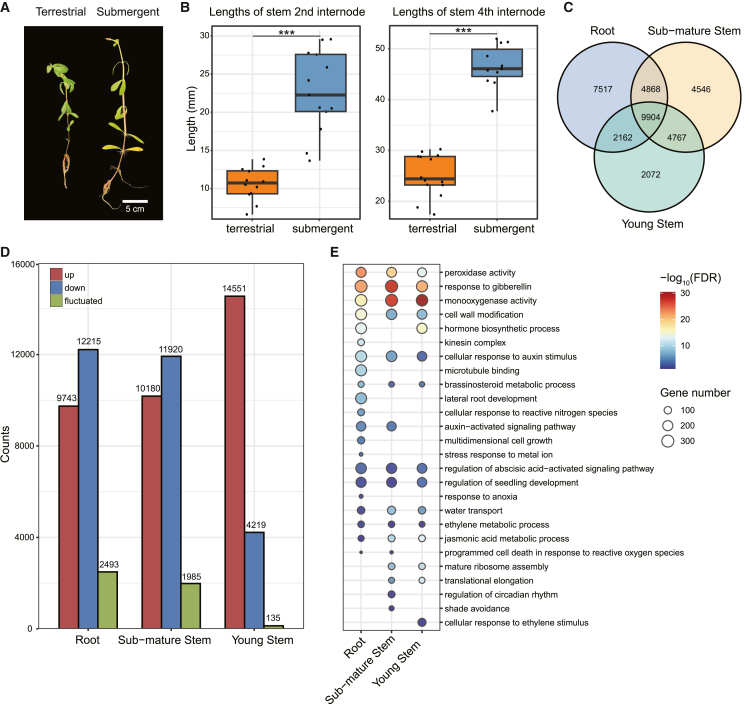
Morphological and transcriptomic variations under submergence in *A. philoxeroides* (A) Pictures of *A. philoxeroides* plants under terrestrial and submergent treatment. The scale bar represents 5 cm. (B) The lengths of 2nd (left) and 4th (right) stem internodes under terrestrial and submergent conditions. *A. philoxeroides* plants were grown for 3 weeks under terrestrial conditions and either maintained under terrestrial conditions or transferred to submergence for 5 days. Data are shown as the mean and SD (∗∗∗*p* < 0.001). (C) Numbers of differentially expressed genes in the three tissues. (D) Numbers of up-regulated, down-regulated, and fluctuated genes in the three tissues. (E) GO enrichment of differentially expressed genes in the three tissues. For each point, the size is proportional to the number of genes, and the colors represent −log_10_(FDR).

The results of the common garden experiments showed that submergence promoted internode elongation and the formation of aerial roots in *A. philoxeroides* (Figures 1A and 1B). Temporal patterns of gene expression under contrasting hydrological conditions were obtained via time-series transcriptome analyses using RNA-seq, which generated 8,356,029,620 clean reads (2,506.81 Gb) that were aligned to the *A. philoxeroides* genome with an average mapping rate of 83.96% (Table S1).

A total of 24,451, 24,085, and 18,905 DEGs were detected in the roots, sub-mature stems, and young stems, respectively. Cumulatively, 35,836 genes were differentially expressed in at least one tissue, and 25,932 genes (72.36%) displayed tissue-specific differential expression. In contrast, a smaller subset of 9,904 genes (27.64%) was differentially expressed across all three tissues (Figure 1C). The number of down-regulated genes in the root and sub-mature stems (11,920 and 12,215, respectively) slightly surpassed their up-regulated counterparts (10,180 and 9,743, respectively). Conversely, the majority of DEGs in young stems (14,551 genes, 76.97%) were up-regulated, with 4,219 being down-regulated (22.32%) (Figure 1D).

Gene Ontology (GO) enrichment analysis revealed the primary functions of DEGs in each tissue (Figures 1E; Table S2). Shared enrichment encompasses processes such as oxidative reactions, water transport, cell wall modifications, and seedling development regulation, along with the metabolism and signaling of various phytohormones, including GA, auxin, brassinosteroid (BR), ethylene, and ABA.^37^ For example, *AphChr09.t496*, which encodes gibberellin receptor GID1B, was differentially expressed in the three tissues. Distinctively, the root-specific DEGs enriched were notably associated with “microtubule binding," “lateral root development,” “cellular response to reactive nitrogen species,” and “metal ion stress,” while the DEGs uniquely detected in the sub-mature stems were significantly enriched for GO terms related to “circadian rhythm” and “shade avoidance.” Additionally, both the roots and sub-mature stems showed significant enrichment of “programmed cell death in response to reactive oxygen species,” indicating a correlation between these biological processes and their respective functions. In the young stems, DEGs of “cellular response to ethylene stimulus” were specifically enriched, including ethylene receptors (*ETR*) *AphChr09*.t590 and *AphChr18.t444*, ethylene insensitive transcription factors 3 (*EIN3*) *AphChr38.t804* and *AphChr45.t609*, and ethylene-responsive transcription factors (*ERF*) *AphChr34.t886* and *AphChr49.t104*.

### Genome-wide identification and characterization of submergence-induced long non-coding RNAs in A. philoxeroides

**Figure 2:**
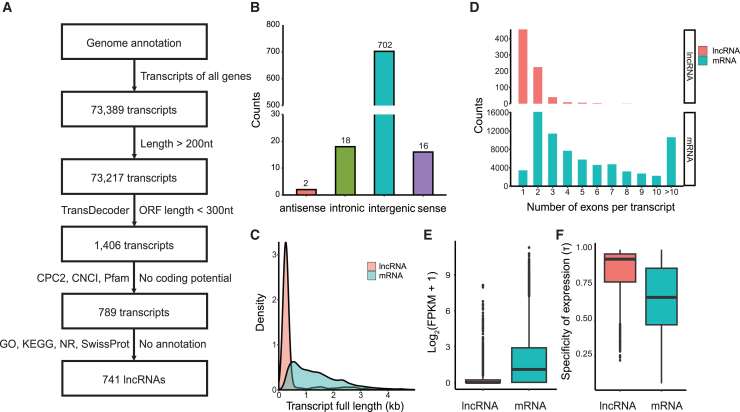
Identification and characterization of lncRNAs in *A. philoxeroides* (A) The pipeline to identify lncRNAs. (B) Number of submergence-regulated lncRNAs. (C) Density distribution of the full length of lncRNAs compared with mRNAs. The X axis represents the full length of transcripts. (D) Number of exons per transcript of submergence-regulated lncRNAs and mRNAs. (E) Average expression log_2_(FPKM +1) of lncRNAs and mRNAs in all samples. (F) Specificity of expression of lncRNAs and mRNAs across all samples. The equation to calculate the specificity index of gene expression (τ) is described in the STAR Methods section.

A total of 741 lncRNAs (Table S3) were identified using the pipeline shown in Figure 2A, including 702 (94.74%) intergenic, 18 (2.43%) intronic, 16 (2.16%) sense, and 2 (0.27%) antisense lncRNAs (Figure 2B). The average length of lncRNAs was 560 nt, which was significantly shorter than that of mRNAs with an average length of 1,460 nt (Wilcoxon test, *p* < 2.2E-16) (Figure 2C). The mean number of exons per lncRNA was also significantly less than that of mRNAs (1.5 vs. 5.9, Wilcoxon test, *p* < 2.2E-16). A large proportion of lncRNAs possessed only 1–2 exons, whereas most mRNAs had more than 2 exons (Figure 2D). The overall expression levels of lncRNAs were significantly lower than those of mRNAs (Wilcoxon test, *p* < 2.2E-16) (Figure 2E). However, lncRNAs exhibited a higher specificity of expression than mRNAs (specificity index 0.84 vs. 0.66, Wilcoxon test, *p* < 2.2E-16) (Figure 2F). A total of 141 lncRNAs were found to be significantly differentially expressed (DELs) between the submerged and terrestrial conditions (fold-change >2 and adjusted *p*-value <0.05) (Figures S2), suggesting their potential roles in response to submergence stress. The results of conservation analysis by searching against publicly available lncRNA databases revealed that only a small proportion (54, 46, 84, and 6) of *A. philoxeroides* lncRNAs showed detectable similarity to the known lncRNAs in PLncDB, CANTATAdb, NONCODE, and PlncRNADB, respectively. Most of the lncRNAs identified in *A. philoxeroides* lacked sequence conservation across plant species.

### Identification and functional predictions of *cis*-regulatory long non-coding RNA-target gene pairs

DEGs located within 100 kb upstream or downstream of DELs were identified as *cis*-targets in this study and were further confirmed by co-expression analysis with a Spearman’s correlation coefficient >0.6 or < − 0.6 and *p* < 0.05. Comprehensive inference of *cis*-regulatory pairs on a genome-scale resulted in the identification of 116, 139, and 102 *cis*-regulatory lncRNA–mRNA pairs in the roots, sub-mature stems, and young stems, respectively (Table S4). Of them, 16 *cis*-targeting lncRNA-mRNA pairs were confirmed by the result of lncRNA-target inference using LncTar, which predicts lncRNA-mRNA interactions based on thermodynamic stability, using the normalized binding free energy (ndG) as a key criterion (Table S5).

**Figure 3:**
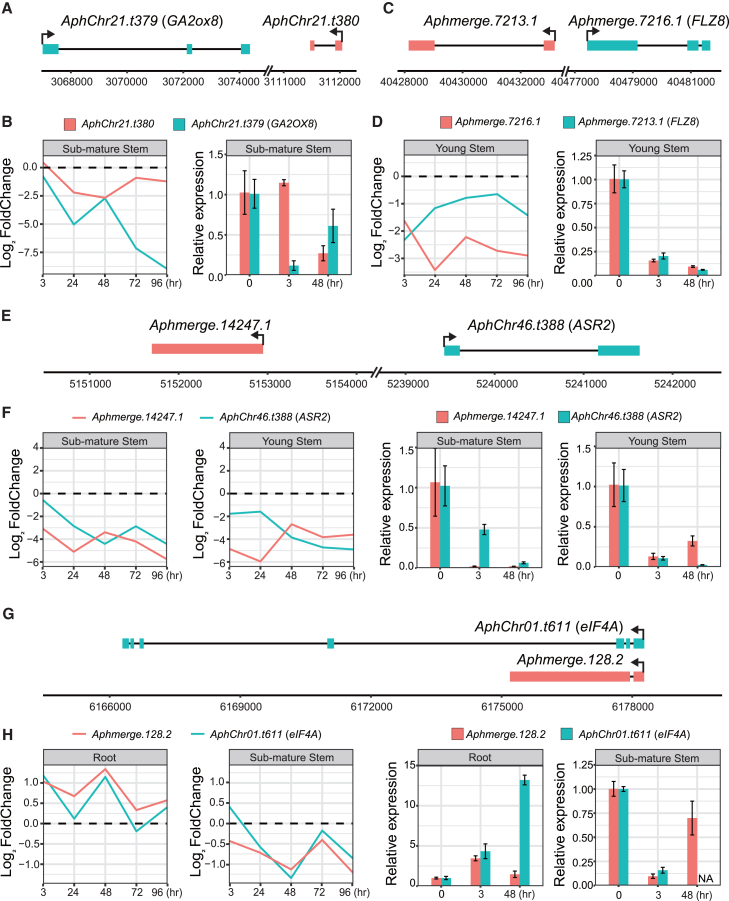
Examples of putative *cis*-regulatory lncRNA-mRNA pairings (A) Positions of lncRNA *AphChr21.t380* and its target gene *AphChr21.t379* (*GA2ox8*). (B) Expression patterns of *AphChr21.t380* and *AphChr21.t379* by RNA-seq (left) and their relative expression (mean and SD) using qRT-PCR (right) in the sub-mature stems. (C) Positions of lncRNA *Aphmerge.7213.1* and its target gene *Aphmerge.7216.1* (*FLZ8)*. (D) Expression patterns of *Aphmerge.7213.1* and A*phmerge.7216.1* by RNA-seq (left) and their relative expression (mean and SD) using qRT-PCR (right) in the young stems. (E) Positions of lncRNA *Aphmerge.14247.1* and its target gene A*phChr46.t388* (*ASR2*). (F) Expression patterns of A*phmerge.14247.1* and *AphChr46.t388* by RNA-seq (left) and their relative expression (mean and SD) using qRT-PCR (right) in the sub-mature and young stems. (G) Positions of lncRNA *Aphmerge.128.2* and its target gene *AphChr01.t611* (*eIF4A*). (H) Expression patterns of *Aphmerge.128.2* and A*phChr01.t611* by RNA-seq (left) and their relative expression (mean and SD) using qRT-PCR (right) in the roots and sub-mature stems.

Functional annotation showed that the *cis*-targets of lncRNAs were mostly associated with phytohormone homeostasis and signaling, transcriptional regulation, and protein translation and degradation (Table S4). For instance, the expression of *AphChr21.t379*, annotated as *gibberellin 2-oxidase 8* (*GA2ox8*), was potentially regulated by the lncRNA *AphChr21.t380* (Figures 3A and 3B), participating in the deactivation of bioactive GAs.^39^ In the sub-mature stems, both *AphChr21.t379* (*GA2ox8*) and lncRNA *AphChr21.t380* were down-regulated upon the submergence treatment, leading to an elevation in bioactive GA levels and contributing to plastic internode elongation in response to submergence stress.^40^ Additionally, the expression of the lncRNA *Aphmerge.7216.1* and the *FCS-like zinc finger 8* (*FLZ8*) gene decreased in young stems (Figures 3C and 3D), which is crucial for sustaining ABA signaling.^41^ The lncRNA *Aphmerge.14247.1* may also be involved in regulating ABA signaling by modulating the expression of *AphChr46.t388*, which encodes abscisic acid-stress-ripening protein 2 (ASR2),^42^^,^^43^^,^^44^ and was observed to be down-regulated in both sub-mature and young stems (Figures 3E and 3F).

Another illustrative case involves lncRNA *Aphmerge.128.2* and its target gene *AphChr01.t611*, a homolog of *eukaryotic initiation factor-4A* (*eIF4A*), which plays an important role in regulating plant growth and development, as well as in responses to abiotic stresses.^45^^,^^46^ The *Aphmerges.128.2*-*eIF4A* transcripts shared a common transcription start site but exhibited contrasting expression patterns across various tissues in a synchronized manner: up-regulated in roots and conversely down-regulated in sub-mature stems (Figures 3G and 3H). This distinction may be related to the varied plastic growth of roots and stems under submerged conditions. The expression patterns of the *cis*-regulatory lncRNA-target gene pairs were confirmed using qRT-PCR (Figures 3B–3D, 3F, and 3H).

### Prediction and functional analyses of *trans*-target genes of long non-coding RNAs

Potential *trans*-regulatory lncRNA–mRNA interactions were detected based on co-expression networks (Table S6). Modules constructed with WGCNA were selected for subsequent analyses based on two criteria: (i) displaying a significant correlation with the submergence treatment with an eigengene significance magnitude greater than 0.3 and a *p* < 0.05; and (ii) containing lncRNA nodes co-expressed with mRNA nodes at a correlation weight exceeding 0.2. This approach led to the detection of 88, 78, and 61 lncRNAs co-expressed with 17,016, 5,731, and 8,804 mRNAs in the roots, sub-mature stems, and young stems, respectively (Table S7). LncTar predicted 10,386 *trans-*acting lncRNA-mRNA pairs as high-confident pairs (Table S8).

Five modules were identified in the roots. Genes included in “blue” and “brown” modules generally decreased in expression, while the “turquoise” module genes were predominantly up-regulated. The “black” module genes exhibited down-regulation at 48 h post-submergence; inversely, the “green” module genes were up-regulated at 24 h (Figure S3). A total of six modules were identified in the sub-mature stems: the “tan,” “turquoise,” and “yellow” module genes were up-regulated, whereas the “blue” and “brown” module genes were down-regulated (Figure S4). Among the seven modules identified in the young stems, the genes included in “blue” and “brown” modules generally showed up-regulation; the “green” and “tan” module genes seem to be activated particularly at 3 h, 24 h, and 72 h post-treatment; and the genes of “turquoise,” “yellow,” and “red” modules were mostly down-regulated (Figure S5).

**Figure 4:**
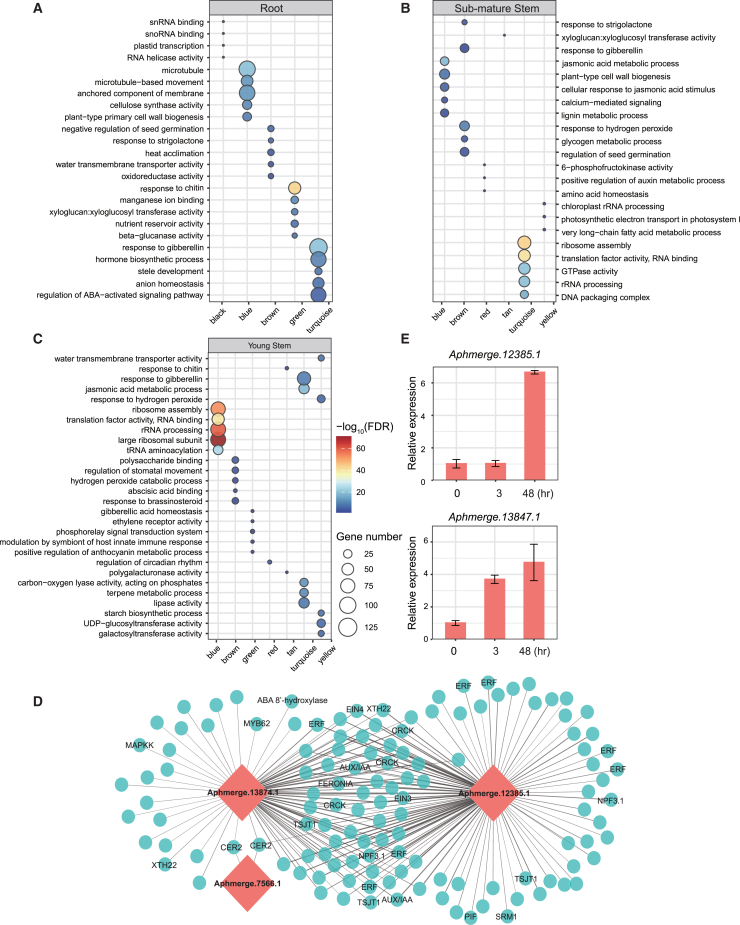
Function enrichments of *trans*-target genes of lncRNAs predicted by co-expression patterns (A–C) GO enrichments of *trans*-target genes of lncRNAs in co-expression modules in the roots, sub-mature stems, and young stems. (D) LncRNAs and their co-expressed mRNAs in the “green” module in the young stems. Red diamonds, lncRNAs; cyan circles, mRNAs. The widths of edges are proportional to the weights. (E) Validation of expression patterns of hub lncRNAs using qRT-PCR. Data are shown as mean and SD.

GO enrichment analyses highlighted the multifaceted roles of *trans*-targets in regulating primary and secondary metabolism, stress responses, phytohormone biosynthesis, homeostasis, and signaling (Figures 4A–4C; Table S9). The down-regulated target genes in the roots were significantly correlated with RNA metabolism, microtubule dynamics, and water transmembrane transporter activity. In contrast, the up-regulated genes were associated with nutrient reservoir activity and hormone biosynthetic processes (Figure 4A). Jasmonic acid (JA) metabolism and responses in the sub-mature stems were highly representative of the down-regulated target genes. Conversely, up-regulation was evident in target genes participating in RNA processing and protein translation (Figure 4B). Down-regulated target genes in the young stems showed connections to the JA metabolic process, starch biosynthetic process, and water transmembrane transporter activity, whereas up-regulated target genes were predominantly involved in RNA processing and translation, as well as signaling of BR, GA, and ethylene (Figure 4C). Specifically, several lncRNAs (*Aphmerge.12385.1*, *Aphmerge.13847.1*, and *Aphmerge.7566.1*) were co-expressed with ethylene-responsive transcription factor (*ERF*), ethylene insensitive 3 (*EIN3*) and *EIN4*, phytochrome interacting factor (*PIF*), and other genes associated with stems elongation growth in the “green” module of the young stems (Figures 4C and 4E), displaying the expression pattern of being activated from the beginning of the submergence treatment, and followed by an up-regulation trend with slight fluctuation and a sharp rise at 72 h (Figure S5). The relative expression of lncRNAs *Aphmerge.12385.1* and *Aphmerge.13847.1* was validated using qRT-PCR, which showed an increase in transcription after submergence, consistent with the RNA-seq results (Figures 4D and S5).

### Validation of long non-coding RNA-target mRNA pairs by *in vivo* over-expression and qRT-PCR

**Figure 5:**
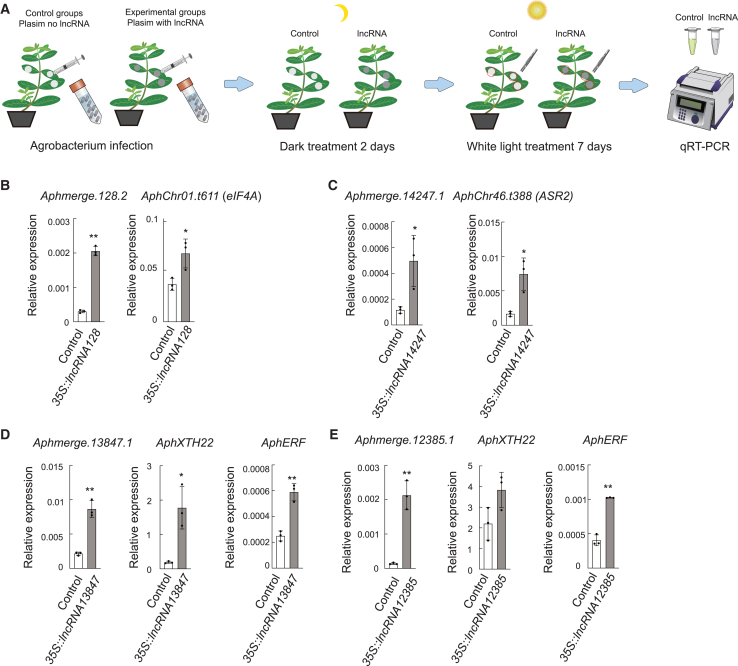
Validation of lncRNA-mRNA target pairs by *in vivo* over-expression and qRT-PCR (A) Diagram of the experiment. (B) Over-expression of lncRNA *Aphmerge.128.2* and relative expression of its putative *cis*-target *AphChr01.t611* (*eIF4A*). (C) Over-expression of lncRNA *Aphmerge.14247.1* and relative expression of its putative *cis*-target *AphChr46.t388* (*ASR2*). (D) Over-expression of lncRNA Aphmerge.13847.1 and relative expression of its putative *trans*-target *AphXTH22* and *AphERF*. (E) Over-expression of lncRNA *Aphmerge.12385.1* and relative expression of its putative *trans*-target *AphXTH22* and *AphERF*. For B-E, data are shown as mean and SD (Student's t-test ∗P < 0.05, ∗∗P < 0.01).

To validate the regulatory roles of lncRNAs on *cis*- and *trans*-target genes, we transiently expressed lncRNA128, lncRNA14247, lncRNA13847, and lncRNA12385 driven by the 35S promoter in *A. philoxeroides*, and quantified their effects on target gene expression via qRT-PCR. The results demonstrated that the over-expression of lncRNA128 and lncRNA14247 significantly upregulated their respective *cis*-target genes *AphChr01.t611* (*eIF4A*) and *AphChr46.t388* (*ASR2*), whereas the transient expression of lncRNA13847 and lncRNA12385 markedly enhanced the transcription levels of their *trans*-target genes *AphXTH22* and *AphERF* (Figure 5).

## Discussion

Phenotypic plasticity allows organisms to adapt to changing environments rapidly by producing distinct phenotypes, enhancing their survival and reproductive success.^47^ As a consequence of global climate change, extreme hydrological events, such as catastrophic floods and droughts, are becoming more frequent and severe.^48^^,^^49^ Flooding imposes multiple stressors on plants, including restricted access to oxygen and carbon dioxide, reduced light availability, nutrient deficiencies, and increased osmotic stresses.^50^ *A. philoxeroides* is an excellent model for studying phenotypically plastic responses to flooding stress because of its remarkable submergence escape capacity by fast stem elongation and extensive pith cavity development upon submergence.^37^^,^^38^^,^^51^^,^^52^^,^^53^

Phenotypically plastic responses take place mostly by altering gene expression and eventually altering ontogenetic trajectory in response to environmental variation. Time course gene expression analyses conducted in this study revealed transcriptome-wide expression variations associated with submergence in *A. philoxeroides*. Functional annotation of differentially expressed genes provided insights into the signaling pathways and molecular processes potentially involved in submergence-induced phenotypic changes. Consistent with previous studies, several elements associated with the ethylene signaling pathway, including ethylene receptors (ETR), ethylene-insensitive transcription factors (EIN), and ethylene-responsive factors (ERF), were differentially expressed in contrast to hydrological conditions. Ethylene is a key player in plant adaptation to flooding, and the fundamental physiological and molecular mechanisms associated with ethylene-mediated plant responses to flooding stress have been widely investigated.^54^^,^^55^^,^^56^ The differential expression of ETR, EIN, and ERF under contrasting hydrological conditions in *A. philoxeroides* suggests their central role in modulating signaling and metabolic responses, and subsequently promoting plastic development, such as rapid internode elongation and aerenchyma formation, through the intrinsic transcriptional activation of downstream plastic growth-related genes.

In addition to the widely reported ethylene signal-related components, we also detected the submergence-induced differential expression of several light signal components, such as PIFs, which were generally associated with the circadian clock and shade avoidance. PIFs function as negative regulators of light responses by repressing photomorphogenesis.^57^^,^^58^ It is now clear that, besides a shortage in cellular oxygen availability, submerged plants are often exposed to a range of low-light intensities depending on the submergence depth and turbidity.^59^^,^^60^ It has been suggested that responses to submergence are *de facto* responses to low light intensity.^61^ Submerged plants in low light had significantly longer petioles than plants submerged in high light.^62^ Moreover, under low-light conditions, different *Arabidopsis* accessions exhibited significant variations in their submergence tolerance.^63^ These studies together highlight the influence of light on plant plastic growth to cope with submergence. It has also been depicted that both submergence and shade can induce similar acclimation responses in plants, viz. accelerated the elongation of internodes and petioles.^62^^,^^64^ However, it remains unclear whether the similar “escape” responses induced by different environmental stimuli are also functionally the same. Further in-depth comparative studies are needed to understand the complex relationship between environmental signals and the resulting phenotypes, and how plants to regulate plastic submergence responses by coordinating light and submergence-induced ethylene signals.

Epigenetic mechanisms, including the epigenetic and epitranscriptomic roles of lncRNAs, are the key link between environmental cues and changes in gene expression.^65^ In this study, a total of 741 lncRNAs were identified in *A. philoxeroides*, with 141 being differentially expressed under contrasting hydrological conditions. Most of the *A. philoxeroides* lncRNAs exhibited low sequence conservation with those of other plants deposited in public databases, including those that have been shown to be involved in waterlogging/submergence adaptation. Poor interspecies conservation of lncDNA sequences has repeatedly been reported in plants and animals, which severely hinders the identification of lncRNA orthologs and functional interpretation by using traditional sequence comparison methods.^66^^,^^67^^,^^68^^,^^69^ It has been proposed, however, that lack of conservation in sequences does not imbue a lack of function, since the functions of lncRNAs may be preserved for the presence of functional elements essential for lncRNA function, such as RNA-binding motifs or splice sites, that are evolutionary constrained but interspersed with longer and less conserved nucleotide sequences.^66^^,^^67^^,^^68^ Additionally, lncRNA conservation may occur beyond DNA sequences, but is conserved at the structural level or by syntenic position.^70^ Diverse patterns of lncRNA conservation are believed to reflect their mode of action.^71^ Nevertheless, considering the rapid evolutionary turnover underlying lncRNAs,^72^^,^^73^ it can reasonably be expected that a portion of the unmatched lncRNAs identified in this study are virtually *A. philoxeroides*-specific, participating in the development of plastic traits under contrasting hydrological conditions.

Differentially expressed lncRNAs involved in response to waterlogging and submergence have been investigated in different plants. More than five thousand lncRNAs associated with waterlogging stress have been identified in two local wheat cultivars from Yangtze River Basin.^74^ A comprehensive transcriptomic analysis revealed 137 differentially expressed lncRNAs co-expressed with genes enriched in the hypoxia response pathway, and most of the co-expressed lncRNAs were colocalized with previously identified quantitative trait loci associated with waterlogging tolerance in maize.^75^ The transcriptomic analysis has also revealed that lncRNA-mRNA regulatory processes in response to waterlogging were mainly related to MAPK signaling pathway, glutathione metabolism, ubiquitin-mediated proteolysis, and so forth in rye, and the signaling of ethylene-related pathways was not mainly dependent on AP2/ERF and WRKY transcription factors, but on other factors.^76^ Studies conducted on cucumbers have identified lncRNAs with regulatory roles in the expression of specific genes under hypoxic stress, with seventy-one lncRNAs being recognized to be involved in acquiring tolerance to hypoxia.^77^ Research on deepwater rice has demonstrated the miRNAs-lncRNAs-mRNAs modules leading to stem elongation under deepwater stress.^78^ Comparing to previous studies, the current study highlighted the significant roles of lncRNAs in mediating the crosstalk between various phytohormones to respond to submergence stress. Validation through qRT-PCR and *in vivo* transient expression proved the functional relations between the *cis*-acting lncRNA *AphChr21.t380* and its target gene associated with GA signaling, the *cis*-acting lncRNAs *Aphmerge.7216.1* and *Aphmerge.14247.1* and their targets involved in regulating ABA signaling, and several *trans*-regulatory lncRNAs *Aphmerge.12385.1*, *Aphmerge.13847.1*, and *Aphmerge.7566.1* and their interacting partners targeting different components of the ethylene signaling pathway, respectively. lncRNA-mRNA regulatory interplay had a big impact on phytohormone homeostasis upon submergence, thereby impacting growth and plastic development. Moreover, the present study unveiled the potential role of lncRNAs in mediating submergence-induced growth alterations by targeting light signal components, such as PIFs. Previous research on submergence-induced elongation has predominantly focused on hypoxia signaling and acclimation. We here demonstrated a previously unknown regulatory means of plastic submergence responses by coordinating external light and internal hormone signals via lncRNA-mRNA interactions.

Despite the findings mentioned above, there is still a lack of understanding of the regulatory mechanisms of lncRNAs in submergence responses. The studies done before are mostly based on statistical correlations, but on causality discovery-based methods. LncRNAs modulate gene expression at multiple regulatory levels in a variety of ways. Integrated molecular and functional characterization of submergence-responsive lncRNAs is thus needed for dissecting the underlying mechanisms of action and understanding how lncRNAs regulate their target genes for implementing specific biological functions.

In conclusion, based on long-read PacBio sequencing and time-course gene expression profiling, we conducted the first genome-wide survey of lncRNAs in *A. philoxeroides* and identified 141 lncRNAs involved in the submergence response. Functional annotation of *cis*- and *trans*-target genes indicated that lncRNAs likely govern a sophisticated regulatory network, fine-tuning gene expression to coordinate hormone signaling, stress responses, and developmental plasticity, thereby contributing significantly to the plant’s extraordinary ability to adapt to submergence. These findings enhance our understanding of the molecular basis of submergence tolerance and plastic phenotypic variation in *A. philoxeroides*. Future research into the interaction between lncRNAs and their partner genes would provide deeper insights into the regulatory mechanisms governing plant development plasticity to hydrological fluctuations, and contribute to developing crop varieties resilient to both extreme precipitation and drought to deal with the growing challenges of drought-flood abrupt alternation owing to global climate change.

### Limitations of the study

In this article, we demonstrate that some lncRNAs are likely associated with a regulatory network that intertwines gene expression regulation, hormone signaling, stress responses, and developmental plasticity in response to submergence in *A. philoxeroides*. However, this study does not include functional experiments to elucidate the precise molecular mechanisms through which these lncRNAs regulate their target genes and influence the plastic development of the plant in response to submergence. Despite these limitations, our findings indicate that lncRNAs are differentially expressed under submergence in *A. philoxeroides*, underscoring the potential importance of lncRNAs in submergence response and resilience to flooding.

## Resource availability

### Lead contact

Further information and requests for resources and reagents should be directed to and will be fulfilled by the lead contact, Ji Yang (jiyang@fudan.edu.cn).

### Materials availability

This study did not generate new unique reagents.

### Data and code availability

• The transcriptome sequencing data generated in this study were deposited in the CNGB data center (https://db.cngb.org/): CNP0005801. They are publicly available as of the date of publication.
• qRT-PCR data reported and customized codes used in this article will be shared by the lead contact upon reasonable request.
• Any additional information required to reanalyze the data reported in this article is available from the lead contact upon request.

## Acknowledgments

This research was supported by the 10.13039/501100012166National Key R&D Program of China to J. Y. and B. Z. (2021YFC2600102).

## Author contributions

Conceptualization, J. Y. and B. Z.; methodology, B. Z., Q. H., J. Y., and R. Q.; investigation, Q. H., R. Q., W. L., and M. Q.; formal analysis, R. Q. and M. Q.; writing - original draft, R. Q. and C. Y.; writing - review and editing, C. Y., J. Y., B. Z., and Q. H.; funding acquisition, J. Y. and B. Z.; supervision, J. Y. and B. Z.

## Declaration of interests

The authors declare no competing interests.

## STAR★Methods

### Key resources table

**Table:** 

REAGENT or RESOURCE	SOURCE	IDENTIFIER
**Biological samples**		

*Alternanthera philoxeroides* invasive population	Fudan University	N/A

**Critical commercial assays**		

TRIzol	Invitrogen	15596018
Ribo-zero kit	Vazyme	N409-01
R1 RNA Cartridge Kit	BiOptic Inc.	C105110
VAHTS Universal V8 RNA-seq Library Prep Kit for Illumina	Vazyme	NR605-02
All-in-One first strand synthesis master mix (with Dnase)	ShareBio	SB-RT001

**Deposited data**		

RNA-seq data	This paper	CNGB: CNP0005801

**Oligonucleotides**		

Primers used in qRT-PCR	Tsingke Biotech Co., Ltd.	Listed in Table S10

**Software and algorithms**		

LoRDEC	Salmela and Rivals, 2014^79^	http://www.atgc-montpellier.fr/lordec/
CD-HIT	Fu et al., 2012^80^	https://sites.google.com/view/cd-hit
minimap2	Li, 2018^81^	https://github.com/lh3/minimap2
StringTie	Pertea et al., 2015^82^	https://github.com/gpertea/stringtie
HISAT2	Kim et al., 2019^83^	https://github.com/DaehwanKimLab/hisat2
CPC2	Kang et al., 2017^84^	https://github.com/gao-lab/CPC2_standalone
CNCI	Sun et al., 2013^85^	http://www.bioinfo.org/software/cnci
Pfam	Mistry et al., 2021^86^	http://pfam.xfam.org/
GffCompare	Pertea and Pertea, 2020^87^	https://github.com/gpertea/gffcompare
WGCNA	Langfelder and Horvath, 2008^88^	https://cran.r-project.org/web/packages/WGCNA/index.html
Cytoscape	Shannon et al., 2003^89^	https://cytoscape.org/

### Experimental model and study participant details

The invasive population of A. philoxeroides used in this paper was collected in Zhuji, Zhejiang Province, China (120°20′ E, 29°40′ N), and the clonal ramets were subsequently maintained in a greenhouse in Fudan University in Shanghai (121°29′ E, 31°14′ N). Asexually propagated seedlings of uniform size were transplanted into plastic pots (dimensions: upper diameter 16 cm, lower diameter 12.5 cm, height 13 cm), each filled with 1.5 L of a 1 : 1 soil mixture (black soil: sand). These were grown under common garden conditions in the greenhouse for five months prior to two treatments simultaneously. For the submergent treatment, plants were fully immersed in 50 cm-deep water, whereas for the terrestrial control, 1 L of water was provided daily to keep the soil wet but well-drained.

### Method details

#### RNA isolation, library preparation and sequencing

Young stems (the 2nd internodes from the apex), sub-mature stems (the 4th internode from the apex), and roots were collected at 0 h, 3 h, 24 h, 48 h, 72 h, and 96 h after the treatment began. Sampling was conducted with three replicates for every treatment, time point and part of the plants, each replicate containing three individual plants. The harvested samples were cleansed by distilled water, immediately frozen by liquid nitrogen, and stored at −80°C before RNA extraction. Total RNA of A. philoxeroides samples were extracted using TRIzol reagent (Invitrogen, USA). Ribo-zero kit (Vazyme, China) was employed for the depletion of ribosomal RNA. The concentration and quality of RNA were assessed using a Qsep100 and R1 RNA Cartridge Kit (BiOptic Inc., China). 50 ng of rRNA-depleted RNA for each sample was used for library preparation of lncRNA-seq using the VAHTS Universal V8 RNA-seq Library Prep Kit for Illumina (Vazyme, China). Libraries were sequenced on Illumina Novaseq with 2 × 150 bp paired-end reads. Raw reads were filtered by removing low-quality reads and adapters, resulting in clean data.

In preparation for PacBio SMRT Iso-Seq libraries, equal quantities of total RNA from submergent and terrestrial treatment samples were pooled and reverse-transcribed into cDNA separately. Subsequently, two PacBio SMRT libraries were generated and sequenced on PacBio Sequel IIe in Circular Consensus Sequencing (CCS) mode. High-quality reads were extracted from the CCS data to obtain HiFi reads.

#### Transcriptome data analysis and differential expression

PacBio HiFi reads underwent initial processing with IsoSeq tools for barcode, primer, and polyA tail removal, followed by clustering and filtering to retain high-quality sequences. Subsequently, these reads were corrected with LoRDEC^79^ utilizing Illumina RNA-seq data. Redundant sequences were removed using CD-HIT.^80^

The refined third-generation transcriptomes were mapped to the *A. philoxeroides* reference genome using minimap2.^81^ StringTie^82^ was used to merge transcripts from PacBio data and the reference annotation.

For Illumina RNA-seq data, HISAT2^83^ was used to map the cleaned reads onto the *A. philoxeroides* reference genome. Transcript abundance was quantified in terms of counts and Fragments Per Kilobase Million (FPKM) values using StringTie. Differentially expressed transcripts of submergent versus terrestrial treatments in three parts of the plant were identified using DESeq2 R package^90^ with the criteria of |log_2_FoldChange|>1 and adjusted *p* value < 0.05.

#### LncRNA identification, classification, and characterization

LncRNAs were identified by the following steps: 1) transcripts with length >200 nt were selected; 2) TransDecoder was used to predict the open reading frame (ORF) of each transcript, and transcripts with ORF length >300 nt were excluded; 3) transcripts exhibiting protein-coding potential were discarded, as assessed by CPC2,^84^ CNCI^85^ and Pfam database^86^; 4) any remaining transcripts with annotations in GO, KEGG, NR, or SwissProt databases were filtered out. The retained transcripts were designated as lncRNAs.

Categorization of these lncRNAs according to their relative location with protein-coding genes was performed using GffCompare.^87^ Information of mRNA location were input as the reference, while lncRNAs were used as the query to acquire class codes of lncRNAs, with ‘u’ denoting intergenic lncRNA (or long intergenic non-coding RNA, lincRNA), ‘i’ denoting intronic lncRNA, ‘x’ showing antisense lncRNA, and ‘o’, ‘j’, ‘c’, ‘m’ or ‘n’ representing sense lncRNA.^91^

Length, number of exons per transcript, overall expression, and expression specificity of lncRNAs and mRNAs were analyzed using customized scripts. Especially, specificity of expression^92^^,^^93^ was calculated by the following equation:

$$\tau =\frac{N-\frac{\sum_{i=1}^{N}x_{i}}{\max\left(x_{i}\right)}}{N-1}$$

τ denotes the specificity index of gene expression; $x_{i}$ denotes FPKM of the gene in each sample; *N* denotes the number of samples, in this case *N* = 2 treatments × 3 parts × 5 time points = 33.

In order to explore the conservation of the identified *A. philoxeroides* lncRNAs, we performed sequence similarity searches against several publicly available plant lncRNA databases, including CANTATAdb,^27^ NONCODE,^28^ PlncRNADB,^29^ and PLncDB.^30^

#### Target gene prediction and function analysis

For target gene prediction and function analysis, only DELs and DEGs were considered. *Cis*-target genes for DELs were defined as significantly co-expressed (Spearman’s correlation coefficient >0.6 or <0.6 and *p* value <0.05) DEGs located within a 100 kb upstream and downstream of the DELs, according to the previously described method.^94^^,^^95^^,^^96^ To predict *trans*-targets, co-expression networks of DELs and DEGs were constructed using weighted gene co-expression network analysis (WGCNA).^88^ In each module with significant correlation to submergent treatment (|eigengene significance| > 0.3, *p* value <0.05), DEGs connected to DELs with a correlation weight >0.2 were considered as targets of the DELs. GO enrichment analyses of target genes were performed to predict function of DELs. LncRNA–mRNA co-expression networks were visualized using Cytoscape.^89^

LncTar^97^ was further used to predict lncRNA target genes. LncTar predicts lncRNA-mRNA interactions based on thermodynamic stability, using the normalized binding free energy (ndG) as a key criterion. We ran LncTar with default settings and applied a threshold of ndG ≤ −0.1, as recommended in the original publication, to ensure reliable predictions.

#### Validation of RNA-sequencing by qRT-PCR

Total RNA was extracted from *A. philoxeroides* sub-mature stems, young stems, and roots under terrestrial and submergent conditions using TRIzol. The All-in-One first strand synthesis master mix (with Dnase) (ShareBio, China) was used to reverse-transcribe RNA into cDNA following the manufacturer’s protocol. Custom-designed specific primers were synthesized by Tsingke Biotech Co., Ltd., China. The cDNAs was then subjected to real-time qPCR using the CFX Connect Real-Time System (Bio-Rad, USA). Each reaction was replicated for 3 times. The relative expression levels were calculated using the 2^−ΔΔCt^ method. The primers used are listed in Table S10.

#### Validation of lncRNA-mRNA pairs by *in vivo* overexpression and qRT-PCR

35S promoter-driven overexpression vectors were constructed, with the experimental and control groups harboring the target lncRNA fragment and empty vectors, respectively, then transformed separately into Agrobacterium *GV3101* competent cells. Overnight cultures of Agrobacterium expressing the lncRNA and control vectors were harvested by spinning, respectively, and the pellets were re-suspended in a solution containing 10 mM MES (2-(N-morpholino) ethanesulfonic acid), 10 mM MgCl_2_, and 200 mM acetosyringone (AS) to a final optical density (OD_600_) of 1.0. This agrobacterium suspension was then injected into each fully expanded leaf of *A.philoxeroides*. After Agrobacterium-mediated transient transformation, RNA was extracted from young plant tissues subjected to 2 days of dark treatment followed by 7 days of white light illumination. Total RNA was extracted from seedlings via the TRIzol (Ambion, 15596018) RNA extraction method. A 2-μg aliquot of RNA was used for first-strand cDNA synthesis with a FastQuant RT kit (Tiangen, KR118-02). Analysis was performed with a Real-Time System CFX96 C1000 Thermal Cycler (Bio-Rad). All experiments were repeated at least three times and independent biological experiments were repeated three times. The *UBC10* gene was used as the internal control. The expression levels of target genes were normalized against the expression of the reference gene UBC10. The relative expression levels were calculated using the 2^−ΔΔCt^ method.^98^ Data are presented as mean values ± SD (*n* = 3, n refers to biological replicates). The significant differences were calculated by Student’s t test (∗*p* < 0.05, ∗∗*p* < 0.01). The primers used are listed in Table S10.

### Quantification and statistical analysis

For Figure 1B, the lengths of 2nd and 4th stem internodes under terrestrial and submergent conditions were measured and analyzed. The following statistical analyses were carried out in RStudio.

For the lengths of the 2nd internodes, the original measurement data comprised submergent (*n* = 13) and terrestrial (*n* = 13) groups. Boxplots were used to identify and exclude any outliers with the upper and lower bounds representing values of 1.5 times the interquartile range from the first and third quartiles, respectively. One outlier was removed from the terrestrial group. Subsequently, normality and homogeneity of variance tests were conducted on the data with the outlier removed. The Shapiro-Wilk normality test yielded *p*-values of 0.4637938 for the submergent group and 0.9443776 for the terrestrial group, both greater than 0.05, indicating normality. The Levene’s test for homogeneity of variance (center = median) gave a *p* value of 0.004828, less than 0.05, indicating heterogeneity of variances. Thus, non-parametric methods were used. The Wilcoxon rank sum exact test (*p* value adjustment method: holm) compared the differences between the submergent and terrestrial groups, resulting in a *p*-value of 2.3e-06.

For the lengths of the 4th internodes, the original data included submergent (*n* = 14) and terrestrial (*n* = 14) groups. Similarly, one outlier was removed from the submergent group. The Shapiro-Wilk normality test yielded *p* values of 0.4475638 for the submergent group and 0.1774463 for the terrestrial group, both greater than 0.05, indicating normality. The Levene’s test for homogeneity of variance (center = median) gave a *p* value of 0.7342, more than 0.05, indicating homogeneity of variances. Thus, two-sample t-test was used to compare the differences between the submergent and terrestrial groups, resulting in a *p*-value of 7.568e-11.

For qRT-PCR, each group was carried out for 3 replicates. The relative expression levels were calculated using the 2^−ΔΔCt^ method^98^ with UBC10 as the reference gene, and the means and SDs (*n* = 3 for each group) were calculated and visualized in RStudio.

## Supplemental information

Document S1. Figures S1–S5

Table S1. Summary of strand-specific Illumina RNA-seq reads

Table S2. GO enrichment of differentially expressed genes in the three tissues

Table S3. LncRNAs identified in this study

Table S4. *Cis*-regulatory lncRNA–mRNA pairs and functional annotation of the *cis*-target genes

Table S5. High-confident lncRNA-mRNA *cis*-pairs predicted via LncTar

Table S6. Parameters of WGCNA for constructing co-expression networks of three tissues

Table S7. Summary of co-expression networks of *trans*-regulatory lncRNA–mRNA pairs

Table S8. Number of high-confident lncRNA-mRNA *trans*-pairs predicted via LncTar

Table S9. GO enrichment of *trans*-target genes of lncRNAs in each co-expression module in three tissues

Table S10. Primers used in qRT-PCR
