# Supplementary material for: Genome-wide identification and functional prediction of long non-coding RNAs in plastic growth in Alternanthera philoxeroides
Source: iScience. 2025 Sep 2;28(10):113488. doi: 10.1016/j.isci.2025.113488 (PMC12475864; doi:10.1016/j.isci.2025.113488)
Supplement: Document S1. Figures S1–S5 [file mmc1.pdf]

**iScience, Volume *28***

## Supplemental information

# Genome-wide identification and functional prediction of long non-coding RNAs in plastic growth in *Alternanthera philoxeroides*

**Ruiyi Qiu, Qianqian Hu, Wenbo Liu, Meiquan Qiu, Chuanwei Yang, Binglian Zheng, and Ji Yang**

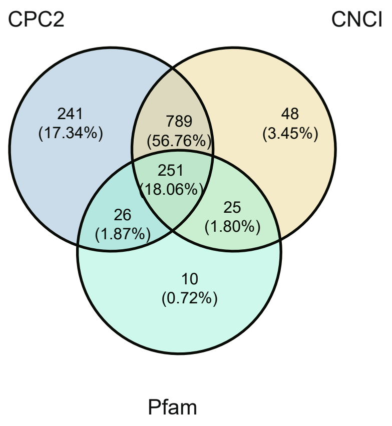


**Figure S1. Venn diagram showing non-coding RNAs identified using CPC2, CNCI, and Pfam.**

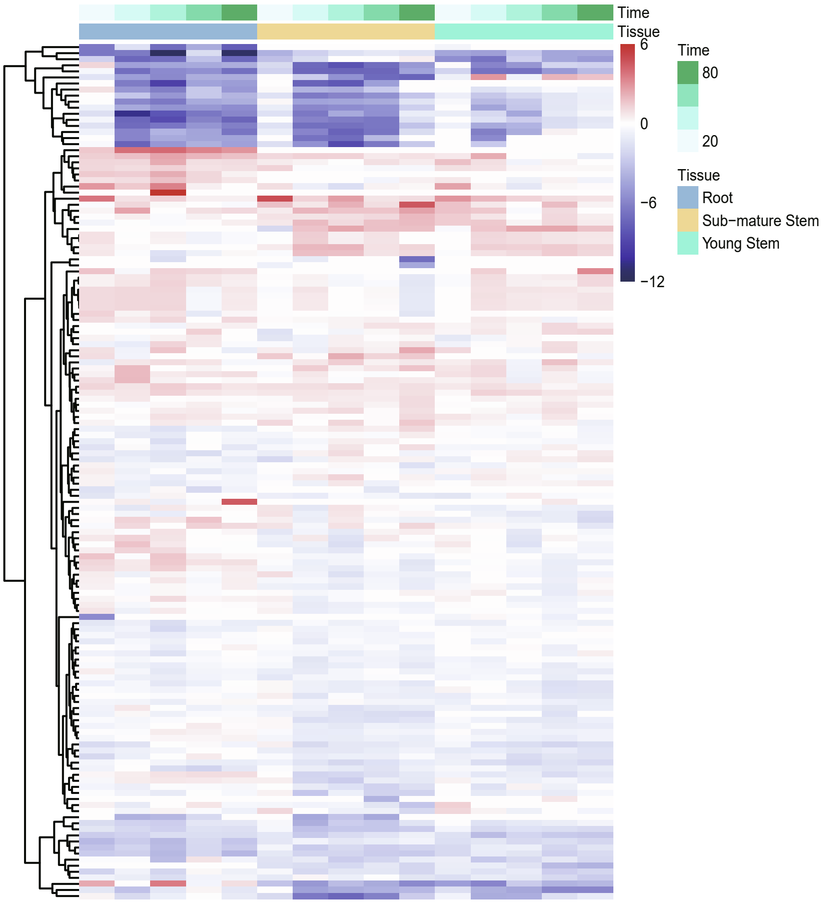


**Figure S2. Heatmap of relative expression of differentially expressed lncRNAs.**
The red and blue rows indicate RNA expression at high and low levels, respectively.

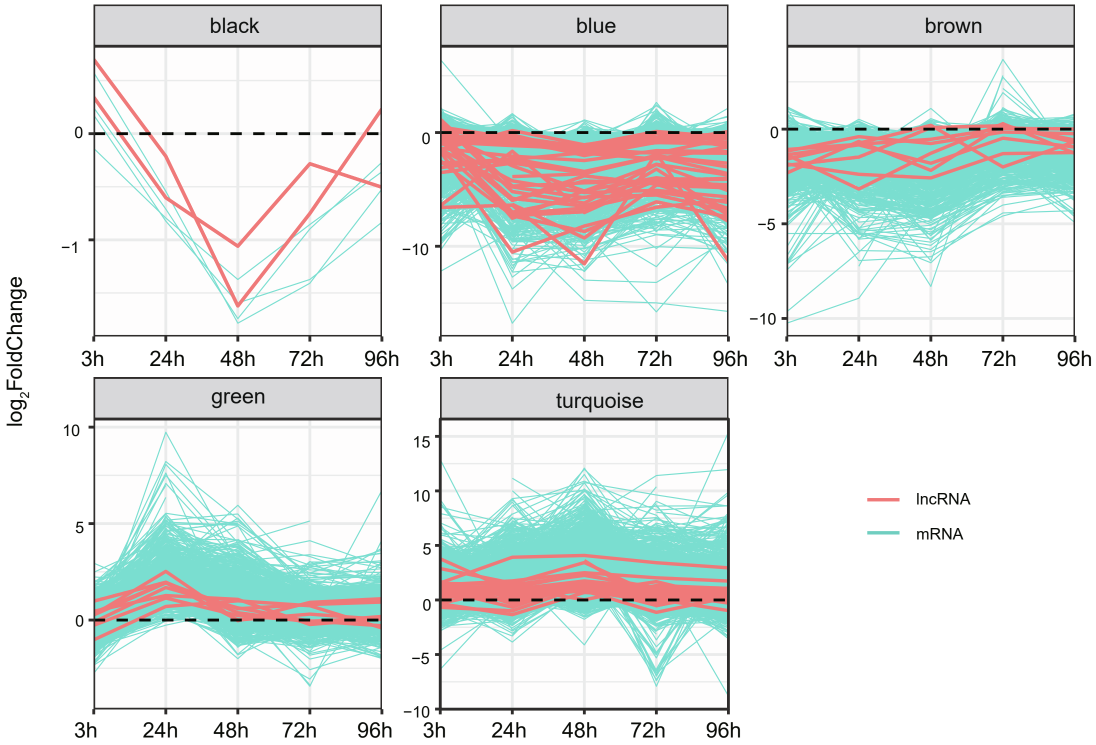


**Figure S3. Line plots showing relative expression dynamics of *trans*-target genes of submergence-responsive lncRNAs in each co-expression module in the roots**.
Thick red lines, lncRNAs; thin cyan lines, mRNAs.

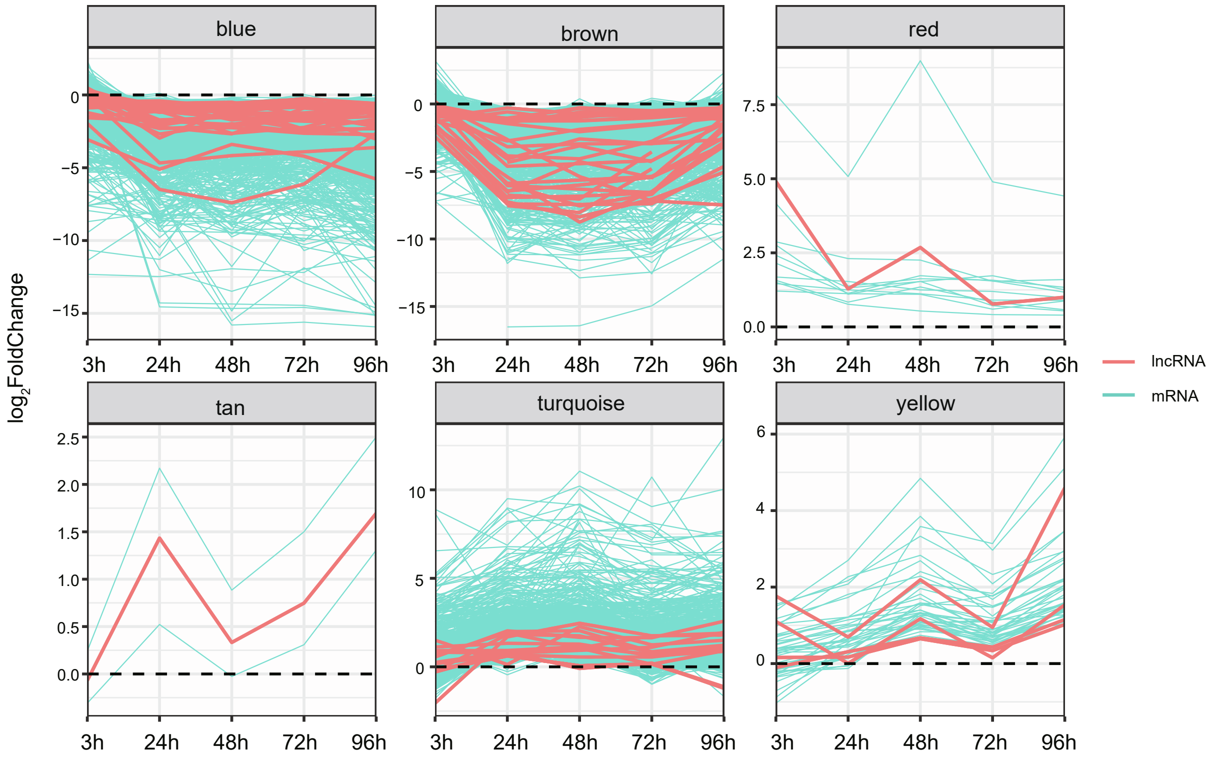


**Figure S4. Line plots showing relative expression dynamics of *trans*-target genes of submergence-responsive lncRNAs in each co-expression module in the sub-mature stems.**
Thick red lines, lncRNAs; thin cyan lines, mRNAs.

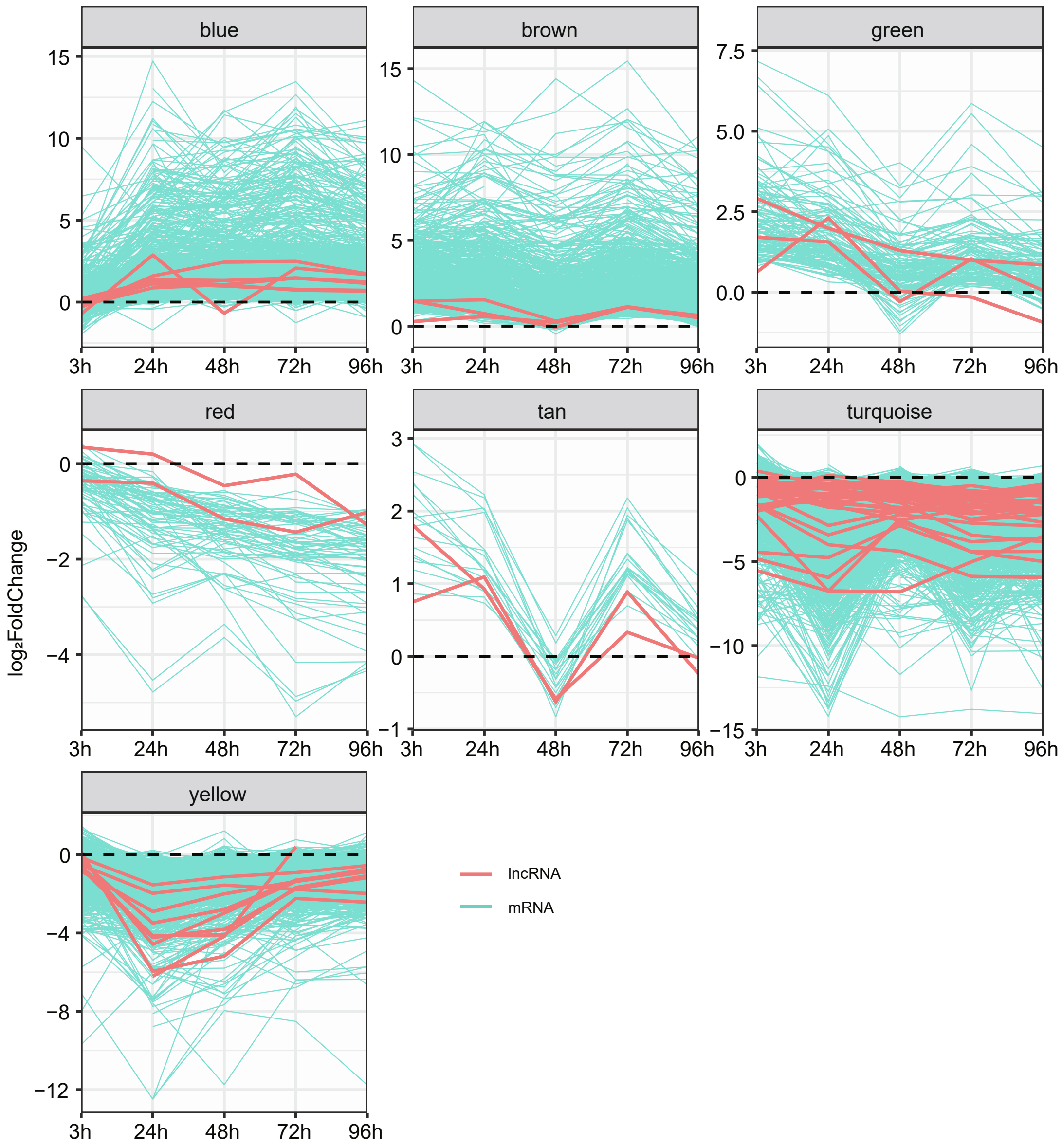


**Figure S5. Line plots showing relative expression dynamics of *trans*-target genes of submergence-responsive lncRNAs in each co-expression module in the young stems.**
Thick red lines, lncRNAs; thin cyan lines, mRNAs.
